# Medical Intelligence and News

**Published:** 1849-01-01

**Authors:** 


### Medical Intelligence and News.

### THE STUDY OF MENTAL DISEASES.

Both in this country and on the continent the study of insanity has recently occupied considerable attention; and additional facilities have been given for obtaining knowledge on this important class of complaints, not only to the general medical student, but to those members of the profession who dedicate themselves exclusively to the treatment of mental diseases. Some years ago, Bethlem and St. Luke's hospitals were made more accessible for such purposes; and reference need only be made to the late proceedings at both these institutions, to show the benefits accruing from these improvements. We are glad, however, to perceive the onward movement proceeds, and that the chief authorities of the first-named noble establishment have just agreed upon important regulations, with the view of increasing the student's means of obtaining instruction, and of diminishing the expense when visiting the wards of Bethlem Hospital. The new rules made by the governors respecting medical pupils, are the following:—

1. That pupils be admitted to attend the hospital practice at Bethlem during two separate terms in every year—the first commencing in October, and terminating in March; the second commencing in April, and terminating in July.

2. That, as soon as practicable, a course of lectures be given in the second term, principally having reference to, and being illustrated by, the cases under consideration at the time.

3. That pupils be permitted to enter for both terms, or either of them, or for attendance on lectures only.

4. That each pupil be required to pay a fee not exceeding five guineas for attendance during each term, or not exceeding two guineas for attendance on lectures only.

5. That each pupil be entitled to attend, and as far as possible have previous notice of, all post-mortem examinations made in the hospital during his pupilage.

6. That the attendance of pupils be at such hours daily as shall be convenient to the medical officers of the establishment, and at the same time least likely to interfere with the duties of those who are students in other hospitals.

7. That the lectures be given from time to time, as shall hereafter be arranged.

If carried out in the spirit actuating the governing body of this royal hospital, much good must follow to all classes, not only of medical men, but to the community at large, by the diffusion of sound and practical knowledge on so important a malady as mental alienation. We say, thanks to the governors, and God speed their benevolent work.

---

### PUERPERAL INSANITY.

*Westminster Medical Society, Saturday, 25th November*, 1848.

Mr. Hird, President, in the Chair.

At this meeting, a paper by John Webster, M.D., F.R.S., was read, entitled "Remarks on the Statistics, Pathology, and Treatment of Puerperal Insanity."

After a few prefatory observations in regard to the severe symptoms frequently characterizing this disease, and the anxiety it often excites amongst relatives and attendants, when they perceive the patient's mind begin to wander, or reason altogether to forsake its seat, Dr. Webster entered into several elaborate yet interesting statements respecting the frequency of puerperal insanity compared with other varieties of mental disease. To illustrate this point, he stated, that in 1091 curable female patients recently attacked by insanity, and admitted into Bethlem Hospital, during the last six years, 131, or one-eighth of the whole, were puerperal cases; thus showing the malady is not so unfrequent as many may perhaps believe. Again, as to the curability of this form of mania, more recoveries were reported than in the other varieties of lunacy; 81 puerperal patients having been cured, or at the rate of 61·83 per cent.; whereas the average recoveries during the last twenty years, in all cases of insane females treated at this institution, was 53·67 per hundred. Hence, three in every five cases of puerperal insanity may be confidently expected to get well within a year. In regard to hereditary tendency to mental disease, 51 of the 131 patients were so predisposed, or 39 per cent.; whilst 41 were suicidal, being at the rate of 31 in every 100. Both these peculiarities are of much importance in this malady, and materially influence the disease, its progress, and result. The total deaths in the 131 puerperal patients amounted to six, or four and a half per cent., thus making the average rate of mortality nearly the same as in other species of insanity, taken collectively. The particulars of the fatal cases, and pathology, next occupied attention, and he (Dr. Webster) stated, that three of the six patients who died were suicidal and hereditary; one was only hereditarily predisposed to insanity, but not suicidal; whilst two, it was reported, had neither of these peculiarities; and none were ever insane previously. In addition to these facts, Dr. Webster also mentioned, that half the deaths occurred in patients who were not affected longer than fifteen days, the shortest period being eleven days, and all were attacked by insanity within seventeen days after their confinement. In none of the dissections were any morbid appearances observed in the abdomen, but the lungs always appeared diseased, as also the brain and membranes. The details of one autopsy were then described, as a specimen of the diseased changes of structure frequently met with in puerperal mania, the principal morbid alterations being, turgidity of the bloodvessels of the brain and membranes; large, bloody points on cutting the cerebral substance; slight serous infiltration of the pia mater, and considerable effusion of fluid in the fifth ventricle; adhesion and purulent ulceration were noticed in the left lung, with hepatization in other portions of that organ, and in the right lung, partial pneumonia in the congestive stage. Although this patient had been delivered only twenty-six days prior to her death, no corpus luteum could be discovered in either ovary, nor any diseased changes of structure in the abdomen. Notwithstanding it appeared rather a digression, the author, in his paper, remarked, that gangrene of the lungs, however rare an occurrence in persons carried off by bodily disease, but without any mental affection, sloughing of that organ was not unfrequent in lunatics. He said so from his own knowledge, and others had also made similar observations, especially in continental asylums for the insane. Dr. Webster afterwards alluded to the treatment of puerperal insanity; and considered cerebral irritation, combined with great exhaustion of the nervous system generally, to constitute the true character of this disease, and that it rarely, if ever, proves inflammatory. He thought depletion, or the use of strong antiphlogistic remedies, became very seldom admissible. Leeches appeared in some cases advisable, but even then should be applied with great caution, and their effects carefully watched. As a general maxim, the author advised the same principles to be followed in the treatment of this malady as in delirium tremens, since the nature of the two diseases were somewhat analogous. Opium, camphor, ammonia, and aromatics, with some of the diffusible stimuli, proved excellent remedies, and ought to be chiefly relied upon. When opium fails to procure sleep, so beneficial in this, as indeed in every form of insanity—then conium, hyoscyamus, or Indian hemp, may be substituted. Mild purgatives, to open the bowels, and sometimes cathartics, should be prescribed; but powerful drastic medicines are seldom advisable. Enemata are also useful, and sometimes with turpentine. When the disease assumes a more chronic form, setons or issues may be made in the neck, &c. The shower-bath, from its strengthening influence, then acts beneficially, whilst tonic remedies, with more nutritious food, become necessary, and prove advantageous; indeed, low diet is very often prejudicial in insane patients, and it has been long remarked in many asylums, that improved nutriment, especially in lunatics who have previously suffered privations, frequently becomes a powerful means for promoting recovery. In recent cases of puerperal insanity, when the circulation is accelerated, accompanied by evident congestion of the brain, leeches to the temples, and behind the ears, or blisters, might then be applied, and afterwards cooling lotions, with ice to the head; whilst tartar-emetic, or ipecacuanha, in nauseating doses, and digitalis, may be administered for the same object. Besides medical treatment, moral means, with judicious occupation and amusements, when proper for the patient, must not be overlooked, as these very often constitute effective adjuncts in the management of the insane. With the view of briefly illustrating the symptoms and treatment proper to pursue under ordinary circumstances, the author next narrated two cases of puerperal insanity, one being affected with mania, the other with melancholia. In the first, or maniacal case, the patient, a single woman, aged twenty-one, whose child did not survive, had hereditary tendency to mental disease, but was reported not suicidal. She was very noisy, incoherent, often much excited, frequently very wild, violent, exceedingly mischievous, used bad language, destroyed her clothes, and paid no regard to personal cleanliness. Took food voraciously, was very restless at night, and dirty in bed. Pulse generally quick, and bowels constipated. The remedies employed consisted of opening medicines, cooling saline mixtures, and croton oil, on one occasion, with regulated diet. Subsequently, bodily occupation and amusements were put in requisition, whereby the patient soon became convalescent. The second case was an example of the variety denominated melancholia. In this patient, a married woman, aged thirty, suicidal and hereditary tendency to mania existed. She was hasty in temper, but naturally cheerful. The attack commenced a month after delivery, and her child was weaned when six weeks old. Had been much debilitated by hæmorrhage after labour; appeared often very depressed; melancholic; generally very desponding of her insane state, and had attempted to injure herself. She took food very unwillingly; could not sleep at night; would scarcely remain in bed, and endeavoured to escape from her room. Pulse of natural frequency, and bowels regular. Early in the disease, leeches were once applied to the temples, and afterwards blisters to the neck on three occasions. Opiates and camphor were prescribed, with purgatives, especially the compound decoction of aloes. Latterly, the cold shower-bath and tonic medicines were employed. The diet, at first light, was subsequently more nutritious, with malt liquor, by which means, and proper occupation conjoined, as the patient improved, with amusements, she recovered. In concluding his paper, of which the above report is merely an abstract; the author made a few observations respecting the employment of restraint to persons labouring under lunacy in any form. Dr. Webster is decidedly opposed to the use of such severe measures; and said, if improper in ordinary cases of mania, mechanical coercion was even more inapplicable to puerperal insanity; and wherever the straight-waistcoat is adopted, lest the patient might injure herself—the excuse commonly assigned by attendants—the exasperation and excitement then exhibited appear more frequently a consequence of, than a warrant for, such barbarous proceedings. This is found to be especially true, in respect of suicidal patients; since experience amply demonstrates, that the mechanical restraint of insane persons so disposed, and even of individuals who have never shown any propensity of the kind, often acts as a highly exciting cause of suicide. The degradation which even lunatics feel, when thus treated like criminals, frequently produces most injurious effects upon the weakened mind of the sufferer; and if the insane patient, subjected to such cruel treatment, be a female of delicate constitution, susceptible feelings, high accomplishments, and of education, the objections to straight-waistcoats, or similar means, become much stronger; as the results, in all likelihood, will prove more disastrous.

Dr. Murphy was of opinion that the antiphlogistic treatment, in cases of puerperal insanity, was very dangerous. He had found opium, where nervous power was deficient, a valuable stimulant. Cases which occur during lactation he considered very fatal. He briefly alluded to puerperal fever being sometimes mistaken for this disease. Puerperal insanity, he stated, was rarely seen in lying-in hospitals, one case in 1000 being about the average to the women delivered in the Dublin Lying-in Hospital. He concluded his remarks by asking Dr. Webster whether he considered that lactation favoured the development of this disease?

Dr. Lankester was decidedly averse to an antiphlogistic treatment in this disease, which he considered closely allied to cholera, epilepsy, and hysteria, in its general characters. He further added, that however well diseases formerly bore bloodletting, he considered that the present type of diseases strongly contra-indicated its use.

Mr. Hilton had had recourse to bloodletting, in these cases, upon the recommendation of the late Dr. D. Davis, and had found it of great service in the case of a poor woman, who was bled copiously on two separate occasions, and who had had administered to her active purgatives, and calomel and opium.

Dr. Skiers considered bleeding of great service where the lungs were healthy. Esquirol advised venesection.

Dr. Webster, in reply, stated that diseases in the present day rarely require the use of the lancet; that restraint, in puerperal insanity, was more commonly had recourse to in France than in England; that this disease is more frequent and fatal in the upper than in the lower classes of society; that the suicidal and hereditary cases of insanity are less tractable, and more fatal, than the other forms of this disease; that the melancholic cases are more protracted, and less curable; and that over-lactation is no doubt a frequent cause of insanity, which, however, is generally very curable; although the disease arises oftener from lying-in than lactation; it also comes on more frequently after weaning than during suckling. Again, puerperal insanity commonly attacks females from the age of twenty to thirty; mania being its most frequent variety; and three cases out of five generally occur before the fourteenth day after delivery; whilst the danger is diminished the more remote the attack supervenes upon parturition. Formerly, this form of insanity was comparatively less frequent, but more fatal than recently. Dr. Haslam, for instance, reports, that when he resided at Bethlem Hospital, in 1644 insane females admitted, only 85 were puerperal patients, being 5 in 100; and Dr. Burrows records 10 deaths and 1 suicide in 57 cases which came under his observation, or more than quadruple the mortality now mentioned. Further, Dr. Copland states, that 1 case in 8 usually proves fatal. In conclusion, Dr. Webster wished it to be clearly understood, that his statistics had reference to the number of insane patients admitted into Bethlem Hospital, and not to the number of women delivered.—(*From the Lancet.*)

---

### AMENDMENT OF THE LAW OF LUNACY.

The following correspondence, in reference to this important subject, was published in the *Morning Chronicle*:

Society for Promoting the Amendment of the Law.—An ordinary meeting of the members of this Society was held last evening, at their chambers in Regent-street. The chair was taken about half-past eight, by Mr. Serjeant Manning.

The minutes of the preceding meeting were read and confirmed.

Mr. Vansittart Neale then moved, pursuant to notice, that it be referred to the committee on equity, "To consider the Law of Partnership, more especially with reference to joint-stock banks;" and, in so doing, dwelt upon the advantages which, in a commercial country like this, must be derived from the principles regulating the responsibilities of partners in joint-stock undertakings being clearly defined and settled; and it would be an important matter for the committee to consider how far the law, as it stood, operated to encourage capitalists to investment in such undertakings. The object was generally to limit the liability of shareholders to the value of their shares.

Mr. C. Webster seconded the motion.

Some conversation ensued, in which Dr. Shelton Mackenzie, Mr. Headlam, and other gentlemen, took part, and during which reference was made in terms of approval to the French law of partnership *en commandité*, by which the general body of shareholders were liable only to the amount of their shares, but certain principal partners in the concern were liable to the whole amount of their fortunes.

The Chairman observed that the principle was not unknown in this country, and that it prevailed in the mining districts — Cornwall especially.

The motion was then agreed to, and the subject was referred to a committee.

The Law of Lunacy.—Mr. James Stewart then brought up and read the report of the committee on equity, to which the following reference had been made—"To consider the state of the law respecting the confinement of persons alleged to be lunatics."

The chief points of the report may be briefly summed up as follows:—

There were three classes of lunatics which had been the subjects of legislation in this country.

1. Pauper lunatics.
2. Persons found to be lunatics under a commission of the Court of Chancery.
3. Persons not being paupers, and not found to be lunatics by a commission of the Court of Chancery, but placed by medical certificate under restraint in lunatic asylums.

With the first two of these classes the committee did not deal in the report; but they invited the particular attention of the Society to the third, consisting, as it principally did, of persons of moderate means, belonging to the middle classes of society, and not possessing property sufficient to bear the expense of a commission *de lunatico.* In 1843 the total number of lunatics in England and Wales had been computed at 20,000, and in 1847 at 23,000. The number at present was estimated at 30,000. Of these about 5000 belonged to the higher and middle classes, and 18,800 were paupers. The report then recited the provisions of the principal act for the protection of these persons (8 and 9 Vic. cap. 100), by which the Lunacy Commission was made permanent, and then proceeded to remark, with reference to the lunatics comprehended in the third class, as above, that all the persons concerned in the capture and confinement of these alleged lunatics, except the commissioners themselves, might in fact be interested in the arrest and detention of them. Simply on the certificate of two physicians, a person might be condemned to perpetual imprisonment on the charge of insanity. Those physicians were not particular individuals selected and appointed to this function on account of special standing or respectability in their profession, but might be any persons whom the relations, or others moving in the arrest of the alleged lunatic, chose to select; and it might be readily inferred that if any evil motives were operating, individuals of distinguished professional character would not be applied to. The persons, also, by whom the actual capture of the alleged lunatic was effected, were often of a low condition in society. Thus, from the relations or others originally moving to effect the capture of an individual alleged to be insane, one and all of the parties concerned in the act might be interested in the confinement of that individual. The commissioners had done much to check the abuses of the system, but it appeared impossible that their powers of visitation could efficiently extend to all cases, and one was cited in which a person improperly confined had not been liberated, on appeal, until after a detention of ten months. Although, therefore, the services of the commissioners had been highly valuable, the committee were of opinion that something more in the way of legislation might be done—first, as regarded the personal liberty of an individual alleged to be a lunatic ; and secondly, as regarded the management of his property when in confinement. There was evidence to show that persons had been improperly confined on certificates obtained from ignorant and inferior practitioners, at the instance of interested relations or false friends, and executed by low and sordid persons, and that cruelties had been perpetrated upon persons thus improperly confined. The question then was, only, whether there was a sufficient amount of practical evil to warrant further legislation, and the committee had come to the decided conclusion that there was. It was proposed to leave untouched that part of the law which enabled medical men to grant certificates. All the committee objected to was, that that certificate should have the effect of authorizing a perpetual imprisonment without further examination, except by the Commissioners in Lunacy, which might not be of effect until a considerable period after the capture. The committee were of opinion that a further opinion upon an alleged case of lunacy should be given by some person having a judicial character and responsibility, within as short a time after the arrest as possible. The Masters in Lunacy would appear to be the most proper persons for this office ; but if there were objections to that course, it might perhaps be thought advisable to resort to the judges of the county courts, or to the magistrates in sessions, or perhaps to call together a small jury to decide upon such cases. The opinion of the committee, however, was decidedly in favour of placing them within the jurisdiction of some judicial person, upon whom the responsibility of the confinement should rest.

With respect to the management of the property of lunatics of the class referred to, the existing law unfortunately made but little provision. When a case of lunacy was in Chancery, the property was taken possession of by the court, and properly administered ; but there was no safeguard for the possessions of those who had been declared lunatic by medical certificate only ; for although there were clauses in the act giving power to the Master in Lunacy to inquire and report, the committee thought them inadequate to their object ; and the Commissioners in Lunacy had, in fact, already declared them to be practically unavailing in the case of lunatics of small means, but that their property was expended or appropriated as their relations or families might arrange. In many cases, no doubt, the property was properly managed ; but it was obvious that it might be mismanaged, and certain that, in some cases, it had been grossly mismanaged. The property of lunatics of this class had been estimated at a million, representing capital of several millions ; and with respect to the management of such property, the committee recommended a very important alteration in the law, which was, that so soon as the fact of the lunacy should be established, the property of the lunatic should vest in an official committee, and be administered by the Masters in Lunacy for his benefit. The machinery in the office of the Masters in Lunacy was more simple and cheap than that of the Masters in Chancery ; and if the principle of *ad valorem* payment, which had, to a certain extent, been already acted upon, were more completely carried out, and a few other alterations made, the committee believed that the Masters in Lunacy would be a court adapted for all cases, not being those of paupers. And the committee had been informed that one per cent. upon the property would pay the expenses of that court. The advantages of such an alteration would be, that the property of a lunatic would be no longer left to the mercy of his relations, or himself exposed to the consequences of their too often illegal acts, but that such property would be properly superintended and administered.

The next point was the regulation of lunatic asylums, upon which the committee reported that they were not prepared, at present, to recommend the abolition of the institutions which already existed, and the substitution of others under the direct control of the Government, but they advised increased powers of inspection, more official visitation, and greater caution in the granting of licences. They also recommended that arrangements should be made for the regular visitation of the asylums by the clergy, for they regretted to observe that there was at present no provision for the spiritual consolation and assistance of the inmates; and they regarded it as the bounden duty of the State to make such provision; and the principle of it had been recognised in a bill introduced into Parliament last session by the Lord Advocate of Scotland. The committee recommended, therefore, that provision for the regular attendance of a clergyman should be made an indispensable condition on the granting of a licence for an asylum. The committee were of opinion, that regular visits of a clergyman would be found of great assistance in the control of such establishments, inasmuch as the patients would be likely to repose confidence in him, and relate to him the circumstances attending their confinement.

The committee next reported, that sufficient provision was not made for the regulation of houses where only one patient was received. These houses were numerous, especially in the neighbourhood of London; and there was reason to suppose that, in many of them, abuses still existed, which had been abolished in other cases; and it was recommended that regular official visitation should be extended to them.

The committee then recommended that the names &c. of persons employed by the keepers of asylums to effect the capture of lunatics, should be placed upon a register, to be kept by the Commissioners in Lunacy. Also, that a coroner's inquest should be held in every case in which death occurred in a lunatic asylum; and that the residence of the proprietors of asylums on the premises should be made imperative. In these matters the committee, sensible, however, that they had by no means exhausted the subject, or embraced all its branches, believed that the law relating to lunacy might be advantageously altered.

Mr. Stewart concluded by moving that the report be printed, and taken into consideration at a future meeting.

Mr. Miller addressed a few words to the meeting, strongly enforcing the point that all the persons engaged in the act of confining an alleged lunatic might be interested in the prolongation of his delusion, and thought that the report did not go quite far enough in recommending only increased inspection.

Mr. M. D. Hill fully admitted the force of the argument. There was undoubtedly great weight in it, for unfortunately the interest of all these parties pointed the wrong way, as against the lunatic. But then he should like to hear a really practical suggestion for amendment, if any could be made, better than those contained in the report. Should we import a precedent from China, where, he had read, the royal physician received a salary while his patient was well, which was discontinued when he became indisposed [a laugh], and so endeavour to divert the interests of these parties in the other direction, by giving them a handsome bonus on the recovery of the lunatic? The report of the committee was highly interesting. The abuses in these private asylums had been very great; there was no reason to believe that they had been altogether eradicated, and he did not think that the labours of the Society could be better directed than in the investigation of so important a subject.

Mr. Clark cited a case, and went into a short argument to show the necessity for an improved system of managing the property of lunatics of small means.

The report was then ordered to be printed, and the members adjourned to Monday, the 11th of December.

*To the Editor of the Morning Chronicle.*

Sir,—The "Society for Promoting the Amendment of the Law" has lately had under its grave consideration the Law of Lunacy. Your paper of the 14th instant contained a report read by Mr. James Stewart, the secretary of the "committee on equity," to whom this question had been submitted. It is much to be lamented that this Society did not, before they published the result of their deliberations, place themselves in communication with parties who are supposed, by their professional occupations, to be practically conversant with the operation of the law they propose to amend. If they had adopted this very obvious and natural course, the "committee on equity" would have had some reason for self-congratulation. The statement which has been officially published by the secretary of the Society is calculated very much to weaken the authority of a body of men having, I believe, the best objects in view. I propose in this letter to notice a few of the inaccuracies contained in the report; for I think, in justice to those who have had the care of the insane, the public should not be led away from the truth by any statements of an *ad captandum* character. It is inserted in the report, that "simply on the certificate of two physicians, a person might be condemned to perpetual imprisonment on the charge of insanity." Such, I assure you, is not the fact. I think it was the duty of the Society, or of those deputed by them to "get up the case," to obtain accurate information on this point before so seriously committing themselves. No "two physicians" can, of their own authority, commit a person alleged to be insane to an asylum. The Act of Parliament expressly forbids it. Surely the "committee on equity," or Mr. J. Stewart, the secretary, had the curiosity to look over the provisions of the "Act (8 and 9 Victoria, cap. 100) for the Regulation of the Care and Treatment of Lunatics." If they had done so, they would have seen that, coupled with the certificates of two qualified practitioners—not being partners—not being relatives of the parties said to be insane—not being personally interested in his confinement—there must be a printed "order," filled up and signed by the relative or party authorizing the detention of the patient. The certificates are invalid without this "order;" and a party receiving and detaining in his house a patient, on the authority of "two physicians" only, commits a misdemeanor, and is liable to be seriously punished. It may be said, "Oh, any person will sign the order; that is but a trifling part of the proceeding." But, gentlemen, it makes a very material alteration in your statement. You publish only a portion of the truth; let us have the whole of it. This is necessary before being qualified to form a correct judgment of the existing state of the law. The Act of Parliament is expressly framed for the purpose of obviating, in every possible way, the unjust confinement of persons on the ground of insanity. The two medical men who are required to sign the certificates, are to see the patient apart from each other; they are to state, in detail, the grounds for their opinions, to specify the particular delusions or actions of the party which, in their opinion, constitute insanity, and such a degree of insanity as to justify a deprivation of liberty. In addition to this, the party receiving the patient, on the "order" and medical certificates, is required to employ a medical gentleman to examine the patient, and this medical man is required by the law to state his opinion of the case, and to forward to the Commissioners, within a few days, a statement of the bodily and mental condition of the party placed under restraint.* In this way, the public has every guarantee against the unjust detention of persons on the plea of unsoundness of mind. If a conspiracy exists, the relative so giving the "order," the two medical men filling up the certificate, the medical man who sees and examines the patient after his admission, and the proprietor of the asylum, must be parties. Compare these facts with the allegation—the unfair representation of the "committee on equity"—that "simply on the certificates of two physicians a person might be condemned to perpetual imprisonment on the charge of insanity!" I now come to the question of "perpetual imprisonment." Surely, the Commissioners in Lunacy will not feel themselves highly complimented. How absurd is the statement. "Perpetual imprisonment!"—the idea is preposterous. Is it likely that if the medical man required to see the patient is disposed unnecessarily to prolong the confinement of a particular individual, that the Commissioners in Lunacy are likely to sanction such a proceeding? They are compelled to visit all licensed establishments, and to examine each inmate, and to discharge those whom they conceive unjustly detained. They may enter a house any day or hour they think proper, and at night if necessary; they are empowered to examine witnesses on oath; they never give any intimation of their visit, and every precaution is taken to prevent the possibility of a party being improperly confined on the plea of mental incapacity. I would ask the "committee on equity" whether there does not exist such a legal document as a writ of *habeas corpus?* and whether this does not operate against the possibility of a person being "condemned to perpetual imprisonment" on the charge of insanity? I have had, for the last ten years of my life, constant opportunities of becoming practically acquainted with the operation of the law of lunacy; and I can state, having had some hundreds of cases of insanity under my care, that I never saw a medical certificate of insanity which the physician was not fully justified in signing; and more than this, I have never seen a person consigned to an asylum unjustly. I believe this is the experience of most men connected with institutions for the treatment of the insane. Cases may arise in which the most skilful, cautious, and experienced medical men will be deceived; but they are of rare occurrence. I have, during a long period of my life, been in the habit of visiting the principal public and private asylums for the insane in this country, and I never saw a person confined without good and valid reasons.

The "committee on equity" propose, in addition to the two medical certificates, "that it might be advisable, before confining a person on the ground of insanity, to resort to the judges of the county court, or to the magistrates in session, or perhaps call together a small jury to decide upon such cases." The suggestion is altogether impracticable, and must have emanated from a person but little acquainted with the condition of those most frequently consigned to asylums. A party is suddenly seized with a paroxysm of insanity. He is violent, smashing everything within his reach; he may have made an attempt on his own life, or on the lives of those about him. It is essentially necessary, for his own security, and the safety of others, that he should, without loss of time, be placed under surveillance, and be protected against his own insane impulses. Picture yourselves carrying such a case before a "judge of a county court," or a "magistrate in session," or "perhaps a small jury," before taking measures to place the patient in a position where his life would be safe, and where immediate medical measures could be adopted to subdue the maniacal

* If the proprietor of the asylum be a medical man, he is qualified to send in the report.

excitement. The idea is Quixotic! In many of these cases it is a matter of the highest importance to immediately remove the patient from home. The irritation to which a phrensied person would be exposed by bringing him before any judicial tribunal, or "small jury," might possibly endanger his life, and greatly increase the violence of his mental excitement. I am certain it would operate injuriously by greatly retarding his recovery. Even in cases where the symptoms were not so acute, the course proposed would be highly objectionable. It is not to be supposed that a knowledge of insanity comes by intuition, and that a person, because he happens to be "a judge of a county court," or a "magistrate in sessions," or a member of a "small jury," is qualified *ex cathedrâ* to detect the delicate shades of disturbed mind, and to pronounce a satisfactory opinion of the competency of a party to control himself or manage his property. In my time, I have seen a large jury pronounce a person, unequivocally insane, of sound mind and fit to be at large. I should not, therefore, be disposed to trust to the discrimination of the small jury proposed; that jury could not come to a proper decision without hearing evidence—evidence could not be adduced and witnesses examined without counsel—and, altogether, the inquiry would be very complicated, vexatious, irritating, and unnecessary. If this mode of procedure be adopted, it is my firm belief that many valuable lives would be sacrificed. Take the case of a man who, in a fit of despondency, or under the influence of a delusion known only to himself, makes an attempt at suicide. He fails in effecting his object; his mind rallies; his relatives are, of course, anxious about his safety; two medical men examine him, and give it as their opinion that he ought, for a time, to be under surveillance. The patient manifests no obvious indications, apart from the attempt at suicide, of insanity. He is taken before one of the proposed tribunals and examined. The patient talks rationally, can give reasonable answers to any questions proposed—appears calm and free from excitement. He is pronounced unfit for confinement. In an hour after this wise decision, he may be found weltering in his own blood! It may be urged, that I have no right to suppose that such would be the judgment of the tribunal. I know enough of the decisions of juries to feel convinced that a verdict of insanity never can be obtained, unless very strong and conclusive evidence is adduced of the existence of insanity. In cases in which the mind is but slightly impaired, as in incipient insanity, where prompt and early treatment is essential to a recovery, a jury would never pronounce in favour of confinement, particularly if the party alleged to be insane protested against it. I cannot, in whatever light it is viewed, conceive a more mischievous "amendment of the law" than that contemplated by the "committee on equity." It is not my purpose to enter into the consideration of the other portions of the report. I shall merely observe, that the statement put forth exhibits but little knowledge of the subject; and I would humbly suggest, before finally deciding upon what alteration of the law the Society may consider necessary to propose for the sanction of the Legislature, that the "committee on equity" should authorize some member of its body to make himself acquainted with the existing operation of the Law of Lunacy, as a preliminary step to any amendment of that law that in their wisdom they may think proper to propose.*

I have the honour to be, yours obediently,

FORBES WINSLOW, M.D.

Sussex House, Hammersmith, Nov. 15, 1848.

---

* The "committee on equity" makes mention of the alleged cruelties practised in private asylums. May I beg their particular attention to the following passage, which I extract from a work recently published, entitled, "Thoughts on Severe Diseases of the Human

*To the Editor of the Morning Chronicle.*

SIR,—Dr. Forbes Winslow, of the Sussex House Lunatic Asylum, Hammersmith, whose letter on the report of the committee of the Law Amendment Society on the law of lunacy appeared in the *Chronicle* of this day, has somewhat hastily formed erroneous impressions of facts of which his mind will be disabused on the printing of the report, the announcement of which appeared in the *Chronicle* of the 14th instant with your summary.

It was not to be expected that during the fluent reading of the report by Mr. James Stewart, (who, by the way, acted as the chairman, and not as the secretary of the committee from which it emanated,) your reporter should seize with minute accuracy every point of detail; but upon the whole he has given an admirable summary, conveying a just impression of the scope and tenour of the document, and an accurate statement of the principal facts.

Upon that portion of the report, however, which relates to the proceedings to be taken before an alleged lunatic can be confined, the statement is not quite complete. The original report contained the following passage:—" In general no such persons can be legally received in a house so licensed *without a written order* from the person sending him, and the medical certificates of two physicians, surgeons, or apothecaries, in such form as prescribed by the acts; nor can even a single person be legally received or taken charge of in an unlicensed house as a lunatic *without such order* and certificates, unless the person receiving him be one deriving no profit from the charge."

Dr. Winslow dwells at great length on the absurdity of deferring the confinement of the alleged lunatic till a judicial investigation should have taken place; but on reference to your summary he will find that the recommendations of the report went no further than to suggest, that at no great length of time after the patient should be confined the propriety of that course should be determined by a competent tribunal. The passage of your summary is, " It was proposed to leave untouched that part of the law which enabled medical men to grant certificates. All that the committee objected to was, that that certificate should have the effect of authorizing a perpetual imprisonment without further examination, except by the Commissioners in Lunacy, which might not take effect until a considerable time after the caption."

These are the points upon which Dr. Winslow has founded his charge of inaccuracy, as to which I must now leave your readers to judge for themselves. Upon the tone and manner of his letter it is unnecessary for me to make any remark.

I am, Sir, your most obedient servant,

JAMES LEADBETTER, Assistant Secretary.

Society for Promoting the Amendment of the Law,
21, Regent-street, Nov. 18.

---

Body," by E. J. Seymour, M.D. Dr. Seymour acted for a period of eight years as one of the Metropolitan Commissioners of Lunacy, and was in the habit of constantly visiting private lunatic asylums. This distinguished physician, whose practical knowledge of insanity is not exceeded by any living medical man, observes, in his chapter on "Mental Derangement:"—

" I must in common justice premise, that during my period of duty as a Commissioner of Lunacy, I never witnessed one case of cruelty. The greatest care was taken; the most assiduous, nay, the most invidious (to innocent people) inquiries were made. Every suspicious case was examined on evidence, and never any case of cruelty, nor anything like it, occurred."—p. 217.

Since this physician resigned the post of Commissioner, private asylums have been placed under much stricter supervision, and, of course, there exists less opportunity for the alleged "cruelties."

*To the Editor of the Morning Chronicle.*

Sir,—I have no wish to enter into a controversy with Mr. Leadbetter, the assistant secretary of the "Society for Promoting the Amendment of the Law." My reply to his letter shall be brief. I have had no opportunity of seeing the "original report," to which he refers. My inferences were drawn from the admirable summary contained in your columns of the 14th instant. In that summary the following words occur:—"Simply on the certificate of two physicians, a person might be condemned to perpetual imprisonment on the charge of insanity." There was nothing in the summary to qualify this statement. I denied its accuracy. It conveyed an erroneous impression to the public mind. I purposely avoided making any allusion to the reflections cast upon the "respectability" and "special standing" of those members of the profession who are occasionally called upon to certify to a person's insanity, prior to confinement. The parties signing the certificates are, says the report, "not selected and appointed to this function on account of special standing or respectability in their professions." No parties in the medical profession are "specially selected and appointed" for this purpose; the law wisely supposing any qualified practitioner to be competent to form a correct opinion in a case of disease, whether it be of the liver, interfering with its functions—or of the brain and nervous system, deranging the operations of the mind.

As the proposed judicial inquiry was to take place after the "arrest" of the patient, I naturally concluded that it would be preliminary to confinement. But whether the poor patient is to be taken before a "judge of a county court," "a magistrate in sessions," or a "small jury," the day before being transferred to an institution specially devoted to the care and cure of the insane, or "within as short a time after his arrest as possible," it does not in the slightest degree modify my opinion of the mischief that would result from this mode of procedure.

I remain, Sir, your obedient servant,

Forbes Winslow, M.D.

Nov. 20, 1848.

---

### THE LAW OF LUNACY.

(BY THE EDITOR.)

Since the correspondence which immediately precedes this article was sent to press, the members of the "Society for the Promotion of the Amendment of the Law" have met, for the purpose of reconsidering the report of the "Committee on Equity," relative to the proposed alterations in the law of lunacy. On the suggestion of Mr. Lathom Browne, the question was adjourned, for the purpose of affording that gentleman an opportunity of bringing forward certain objections which he considered almost fatal to the contemplated changes. At the request of Mr. Browne, the discussion of the subject was again deferred. At the last meeting of the Society, Mr. Lathom Browne stated in detail, with great ability and much effect, his objections to the report. A long and interesting discussion followed, and the result was, that *the report was unanimously referred back to the Committee.* We congratulate the profession generally, and particularly that portion engaged specially in the treatment of the insane, on the wise determination to which the Society has come. We have—God forbid!—no objection to a judicious amendment of the law of lunacy; our best directed efforts, our untiring energies, we freely offer to the "Society for the Promotion of the Amendment of the Law," if they will submit to the profession any practical suggestion in reference to this important branch of medical jurisprudence. We have no desire to protect those who abuse the important, the sacred trusts, reposed in them. We do not believe that parties connected with institutions for the treatment of the insane have any wish to screen themselves from strict surveillance. All they ask is, that a body of gentlemen, like those connected with the "Society for the Promotion of the Amendment of the Law," should exercise a little more charity towards a number of gentlemen devoted to the study and practice of psychological medicine. "Mad doctors," as they are vulgarly denominated, are not "ogres," "Bluebeards," or other monsters of cruelty, and it is not fair to hold them up to public odium and obloquy. We considered the language of the report anything but fair towards the profession engaged in the treatment of the insane. Its spirit was bad. It was calculated to foster in the public mind prejudices and false impressions against a certain class of practitioners engaged in most arduous, anxious, and responsible duties. The advocacy of truth requires no such weapons. The abuses of bygone days are not to be disinterred for the purpose of exciting or keeping alive in the minds of persons (always ready to traduce the professors of medicine) a prejudice against establishments devoted to the reception of persons upon whom God has lain his afflicting hand, depriving them of the healthy use of one of the noblest attributes of the human mind!

It may not be difficult for the Society to bring forward isolated cases, in which parties have not been humanely or scientifically treated in private lunatic asylums.

The question for consideration is, not whether there do not exist abuses inseparably connected with the present system of managing the insane, but whether they are of *such magnitude as to justify an application to the Legislature for an alteration of the law of Lunacy.* Under the most perfect —as perfect as human legislation can make them—code of laws, gross cases of injustice to individuals will occasionally occur. Innocent persons have been hanged, on the suspicion of murder; on *this account*, certainly, we should not be justified in recommending either the abolition of trial by jury, or of capital punishments! A recent case has come before the public of a gentleman, who was tried, convicted, and transported on the charge of forgery. The Secretary of State for the Home Department has, in consequence of certain facts which have been laid before him, reconsidered the case; and the result is, the party in question has been allowed to return to this country. We would not, for one moment, think of reflecting on the wisdom of the learned and able judge who presided at this trial, nor of the gentlemen who constituted the jury, that returned the verdict of guilty. Far from it. The case was patiently and anxiously investigated, and no blame can attach to any party associated with the legal proceedings. We adduce these cases to establish the point, that, with the most cautious vigilance—the most patient investigation—instances of injustice, cruelty, and injury to individuals may occasionally arise. We ought to legislate on enlarged and philosophical views of what we conceive to be anomalies in the state of society, and not, directly an aggravated case of abuse occurs, run to Parliament for a special legislative enactment. It is our duty to cultivate the habit of looking at the bright as well as the dark side of human nature, and be more ready to hold good and virtuous men up to public approbation, than to denounce those who do not manifest in their conduct all the cardinal virtues.

